# 1917. A Year's Work in the Hospitals and Medical Charities of London

**Published:** 1918-06-22

**Authors:** 


### 1917.

### A YEAR'S WORK IN THE HOSPITALS AND MEDICAL CHARITIES OF LONDON.

### ST. MARYLEBONE AND WEST CENTRAL DISTRICT.

Comprising St. Marylebone, St. John's Wood, Bloomsbury, Holborn, &c.

| No. of Beds. | No. of Beds Daily Occupied. | HOSPITALS. | In-patients. | Out-patient Attendances. | Total Expenditure. | Income. | | | Total Income. | Legacies not included in preceding column. |
|---|---|---|---|---|---|---|---|---|---|---|
| | | | | | | Charitable. | Proprietary. | Patients' Payments. | | |
| | | | | | £ | £ | £ | £ | £ | £ |
| 74 | 54 | French ... ... ... ... | 724 | 18,604 | 9,030 | 6,781 | 2,154 | 1,136 | 10,071 | 4,319 |
| 85 | 73 | Italian ... ... ... ... | 922 | 6,192 | 7,464 | 2,688 | 1,801 | 4,029 | 8,518 | 275 |
| 170 | 155 | London Homœopathic ... ... | 1,621 | 53,030 | 20,504 | 7,415 | 3,671 | 8,260 | 19,346 | 1,200 |
| 172 | 140 | SS. John and Elizabeth ... ... | 1,204 | ... | 17,158 | 1,964 | 9,909 | 7,509 | 19,382 | 3,400 |
| 357 | 312 | The Middlesex ... ... ... | 4,764 | 164,859 | 56,398 | 19,438 | 10,886 | 7,693 | 38,017 | 4,207 |
| 90 | 79 | Alexandra, for Children... ... | 95 | 376 | 5,992 | 3,486 | 963 | 3,068 | 7,517 | 575 |
| 210 | 202 | Hospital for Sick Children ... | 2,792 | 93,652 | 27,033 | 14,517 | 6,629 | 2,594 | 23,740 | 4,693 |
| 24 | 22 | S. Monica's, for Children ... | 137 | ... | 1,871 | 760 | 156 | 377 | 1,293 | 250 |
| 82 | 73 | Queen Charlotte's Lying-In ... | 1,947 | 15,110 | 11,056 | 7,753 | 1,036 | 1,251 | 10,040 | 186 |
| 71 | 62 | Elizabeth Garrett Anderson Hospital ... ... ... | 1,217 | 33,228 | 12,838 | 4,762 | 1,929 | 2,722 | 9,413 | 2,658 |
| 70 | 54 | Samaritan Free ... ... ... | 1,062 | 12,277 | 9,187 | 6,882 | 727 | ... | 7,609 | 399 |
| 207 | 191 | National for the Paralysed, &c.... | 868 | 48,173 | 27,471 | 11,904 | 2 477 | 11,746 | 26,127 | 1,722 |
| 86 | 84 | Hospital for Epilepsy, &c. ... | 419 | 19,942 | 9,594 | 4,651 | 340 | 3,598 | 8,589 | 500 |
| 82 | 75 | West End, for Epilepsy, &c. ... | 372 | 35,849 | 10,075 | 4,897 | 1,770 | 3,433 | 10,100 | 15 |
| 40 | 23 | Central London Ophthalmic ... | 465 | 27,347 | 3,513 | 3,011 | 226 | ... | 3,237 | 4,279 |
| 16 | 14 | Western Ophthalmic ... ... | 401 | 23,620 | 2,445 | 2,370 | 119 | 498 | 2,987 | 1,000 |
| 197 | 186 | Royal National Orthopædic ... | 796 | 41,349 | 20,135 | 6,304 | 1,960 | 12,079 | 20,343 | 1,839 |
| 38 | 31 | Florence Nightingale Home ... | 472 | ... | 5,514 | 2,137 | 331 | 2,838 | 5,306 | ... |
| 49 | 47 | The Middlesex Cancer ... ... | 127 | 562 | 6,628 | 2,589 | 3,079 | ... | 5,668 | 340 |
| 28 | 12 | Metropolitan Ear, &c. ... ... | 368 | 10,605 | 2,414 | 778 | 3 | 1,384 | 2,165 | ... |
| 2,148 | 1,889 | | 20,773 | 604,775 | 266,320 | 115,087 | 50,166 | 74,215 | 239,468 | 31,857 |
| | | DISPENSARIES. | | | | | | | | |
| ... | ... | London Medical Mission ... | ... | 6,289 | 1,289 | 493 | 706 | 222 | 1,421 | ... |
| ... | ... | Margaret Street, for Consumption | ... | 6,881 | 788 | 417 | 509 | 231 | 1,157 | ... |
| ... | ... | St. John's Wood Provident ... | ... | 13,295 | 440 | 271 | 32 | 150 | 453 | ... |
| ... | ... | St. Marylebone General ... ... | ... | 11,070 | 1,408 | 553 | 226 | 396 | 1,175 | ... |
| ... | ... | Western General ... ... ... | ... | 10,462 | 981 | 643 | 178 | 16 | 837 | 72 |
| 2,148 | 1,889 | | 20,773 | 652,772 | 271,226 | 117,464 | 51,817 | 75,230 | 244,511 | 31,929 |

### WESTMINSTER DISTRICT.—Comprising Westminster City and Liberties.

| No. of Beds. | No. of Beds Daily Occupied. | HOSPITALS. | In-patients. | Out-patient Attendances. | Total Expenditure. | Charitable. | Proprietary. | Patients' Payments. | Total Income. | Legacies not included in preceding column. |
|---|---|---|---|---|---|---|---|---|---|---|
| | | | | | £ | £ | £ | £ | £ | £ |
| 300 | 256 | Charing Cross ... ... ... | 3,438 | 81,226 | 39,668 | 27,816 | 4,679 | 10,204 | 42,699 | 1 443 |
| 218 | 198 | Westminster ... ... ... | 1,682 | 61,248 | 30,864 | 10,268 | 6,955 | 7,862 | 25,085 | 605 |
| 160 | 145 | Ventnor, for Consumption ... | 534 | ... | 17,903 | 5,614 | 3,679 | 7,138 | 16,431 | 1,667 |
| 37 | 20 | Grosvenor, for Women & Children | 309 | 5,544 | 3,280 | 1,986 | 493 | 621 | 3,100 | 1,900 |
| 67 | 60 | Hospital for Women ... ... | 996 | 8,601 | 7,715 | 4,827 | 861 | 1,479 | 7,167 | ... |
| 25 | 18 | Gordon, for Fistula ... ... | 330 | 3,283 | 2,291 | 872 | ... | 2,003 | 2,875 | 10 |
| 42 | 25 | National, for Diseases of Heart... | 174 | 17,116 | 5,144 | 2,890 | 450 | 1,505 | 4,845 | 614 |
| 40 | 35 | Male Lock ... ... ... | 435 | 38,668 | 7,153 | 800 | 25 | 4,226 | 5,051 | ... |
| 40 | 31 | Royal Westminster Ophthalmic... | 638 | 31,541 | 4,737 | 2,220 | 1,443 | 594 | 4,257 | 2,450 |
| ... | ... | Royal Dental ... ... ... | ... | 30,326 | 4,149 | 3,655 | 1,175 | 368 | 5,198 | 70 |
| 32 | 28 | St. Peter's, for Stone ... ... | 470 | 31,698 | 5,847 | 2,038 | 593 | 2,265 | 4,901 | 177 |
| 50 | 39 | Infants' Hospital, Vincent Square | 365 | 3,710 | 3,963 | 3,065 | 79 | ... | 3,144 | 100 |
| 40 | 20 | St. John's, for Skin Diseases ... | 164 | 36,302 | 5,261 | 2,265 | 149 | 2,491 | 4,905 | 100 |
| 57 | 32 | Throat Hospital, Golden Square | 878 | 39,737 | 6,735 | 3,020 | 110 | 3,808 | 6,938 | 3,486 |
| 1,108 | 907 | | 10,413 | 389,000 | 144,710 | 71,336 | 20,696 | 44,564 | 136,596 | 12,622 |
| | | DISPENSARIES. | | | | | | | | |
| ... | ... | Public ... ... ... ... | ... | 10,426 | 685 | 332 | 221 | ... | 553 | 20 |
| ... | ... | St. George's, Hanover Square ... | ... | 1,549 | 396 | 247 | 94 | 95 | 436 | ... |
| ... | ... | Western ... ... ... ... | ... | 12,632 | 1,315 | 215 | 580 | 553 | 1,348 | ... |
| ... | ... | Westminster General ... ... | ... | 14,249 | 892 | 344 | 426 | 98 | 868 | 225 |
| 1,108 | 907 | | 10,413 | 427,856 | 147,998 | 72,474 | 22,017 | 45,310 | 139,801 | 12,867 |

### CITY AND EAST CENTRAL DISTRICT.

Comprising the City, St. Luke's, Shoreditch, Finsbury, and Clerkenwell.

| No. of Beds. | No. of Beds Daily Occupied. | Hospitals. | In-patients. | Out-patient Attendances. | Total Expenditure. | Income. | | | Total Income. | Legacies not included in preceding column. |
|---|---|---|---|---|---|---|---|---|---|---|
| | | | | | | Charitable. | Proprietary. | Patients' Payments. | | |
| | | | | | £ | £ | £ | £ | £ | £ |
| 403 | 356 | Metropolitan ... ... ... | 3,169 | 67 591 | 40,535 | 12,610 | 1,781 | 20,894 | 35,285 | 1,448 |
| 175 | 149 | Royal Free ... ... ... | 2,226 | 114,099 | 34,879 | 10,946 | 5,733 | 2,449 | 19,128 | 12,461 |
| 689 | 514 | St. Bartholomew's ... ... | 6,759 | 239,623 | 112,932 | 14,322 | 80,823 | 14,296 | 109,441 | 4,350 |
| 110 | 95 | Royal, for Diseases of the Chest. | 744 | 25,700 | 14,198 | 16,789 | 439 | 9,889 | 27,117 | 1,324 |
| 170 | 162 | Queen's, for Children ... ... | 1,852 | 111,806 | 23,224 | 14,928 | 766 | 1,123 | 16,817 | 4,104 |
| 61 | 52 | City of London Lying-In ... | 1,345 | 5,689 | 8,900 | 3,633 | 2,947 | 1,325 | 7,905 | 200 |
| 11 | 8 | Jewish Maternity Hospital ... | 246 | ... | 1,718 | 1,126 | 58 | 496 | 1,680 | 167 |
| 52 | 42 | St. Mark's, for Fistula ... ... | 657 | 4,333 | 7,033 | 4,322 | 1,347 | 24 | 5,693 | 1,852 |
| 138 | 112 | Royal London Ophthalmic ... | 2 255 | 109,364 | 18,433 | 10,339 | 2,305 | 2,971 | 15,615 | 5,802 |
| 43 | 40 | Central London Throat and Ear. | 616 | 39,937 | 6,284 | 1,536 | 435 | 4,537 | 6,508 | 250 |
| 1,852 | 1,530 | | 19,869 | 718,142 | 268,136 | 90,551 | 96,634 | 58,004 | 245,189 | 31,958 |
| | | **Dispensaries.** | | | | | | | | |
| ... | ... | Billingsgate Medical Mission ... | ... | 8,018 | 717 | 517 | ... | 76 | 593 | ... |
| ... | ... | City ... ... ... ... | ... | 12,532 | 871 | 824 | 114 | ... | 938 | ... |
| ... | ... | Farringdon General ... ... | ... | 5,484 | 506 | 330 | 15 | 152 | 497 | ... |
| ... | ... | Finsbury ... ... ... ... | ... | 27,285 | 1,250 | 309 | 648 | 218 | 1,175 | ... |
| ... | ... | Metropolitan ... ... ... | ... | 2,350 | 643 | 238 | 327 | 75 | 640 | ... |
| ... | ... | Royal General ... ... ... | ... | 8,095 | 726 | 220 | 398 | 59 | 677 | ... |
| 1,852 | 1,530 | | 19,869 | 781,906 | 272,849 | 92,989 | 98,136 | 58,584 | 249,709 | 31,958 |

### ISLINGTON AND NORTH-WEST DISTRICT.

Comprising Islington, Holloway, Highbury, Hampstead, Highgate, St. Pancras, Stoke Newington, Tottenham, &c.

| No. of Beds. | No. of Beds Daily Occupied. | Hospitals. | In-patients. | Out-patient Attendances. | Total Expenditure. | Income. | | | Total Income. | Legacies not included in preceding column. |
|---|---|---|---|---|---|---|---|---|---|---|
| | | | | | | Charitable. | Proprietary. | Patients' Payments. | | |
| | | | | | £ | £ | £ | £ | £ | £ |
| 393 | 351 | Great Northern Central ... ... | 3,824 | 104,667 | 44,588 | 22,168 | 2,008 | 19,854 | 44,030 | 6,605 |
| 137 | 110 | Hampstead General Hospital ... | 1,080 | 32,670 | 16,830 | 8,373 | 902 | 4,582 | 13,857 | 574 |
| 120 | 97 | London Temperance ... ... | 1,156 | 66.220 | 12,590 | 8,159 | 2 213 | 2,444 | 12,846 | 15,475 |
| 125 | 98 | Tottenham (Prince of Wales's)... | 1,876 | 81,878 | 15,143 | 13,223 | 878 | 194 | 14,295 | 2,667 |
| 492 | 449 | University College ... ... | 4,973 | 131,746 | 49,186 | 20,255 | 8,615 | 17,971 | 46,841 | 1,758 |
| 110 | 104 | Mount Vernon, for Consumption | 315 | 16,218 | 14,565 | 3,154 | 4,040 | 5,728 | 12,922 | 1,000 |
| 20 | 18 | Children's Home Hospital, Barnet | 30 | ... | 682 | 549 | 262 | 59 | 870 | ... |
| 160 | 64 | London Fever ... ... ... | 1,318 | ... | 14,412 | 6,447 | 2,087 | 5,728 | 14,262 | 1,116 |
| 22 | 3 | Bushey Heath Cottage ... ... | 60 | ... | 770 | 249 | 83 | 64 | 396 | ... |
| 25 | 20 | Enfield Cottage ... ... ... | 233 | ... | 1,648 | 993 | 286 | 209 | 1,488 | 205 |
| 23 | 16 | St. Saviour's Hospital ... ... | 144 | ... | 2,518 | 1,262 | 230 | 1,067 | 2,559 | 100 |
| 48 | 40 | St. Columba's Hospital ... ... | 150 | ... | 6,108 | 4,193 | 471 | 457 | 5,121 | 2,700 |
| 72 | 56 | Willesden Cottage ... ... | 555 | 1,031 | 4,222 | 1,507 | 189 | 3,011 | 4,707 | 522 |
| 30 | 17 | Wood Green Cottage ... ... | 295 | 63 | 1,945 | 1,084 | 89 | 521 | 1,694 | ... |
| 20 | 20 | Santa Claus Home ... ... | 24 | ... | 1,183 | 1,031 | 128 | 87 | 1,246 | ... |
| 26 | 11 | Stoke Newington Home Hospital | 120 | ... | 1,457 | 745 | 389 | 278 | 1,412 | ... |
| 56 | 53 | Hospital for Incurable Children | 30 | ... | 2,687 | 777 | 710 | 1,107 | 2,594 | 545 |
| 18 | 18 | Winifred House, Holloway ... | 17 | ... | 1,045 | 534 | 103 | 227 | 864 | 477 |
| 20 | 20 | Highgate Convalescent Home ... | 121 | ... | 647 | 511 | 129 | 11 | 651 | ... |
| 80 | 72 | St. Andrew's, Dollis Hill ... | 442 | ... | 7,815 | 1,885 | 926 | 5,460 | 8,272 | 27 |
| 1,997 | 1,637 | | 16,763 | 434,493 | 200,041 | 97,100 | 24,768 | 69,059 | 190,927 | 33,771 |
| | | **Dispensaries** | | | | | | | | |
| ... | ... | Child's Hill Provident ... ... | ... | 3,900 | 88 | 7 | 8 | 73 | 88 | ... |
| ... | ... | Hampstead Provident ... ... | ... | 12,260 | 770 | 226 | 60 | 519 | 805 | ... |
| ... | ... | Islington ... ... ... ... | ... | 41,882 | 839 | 157 | 81 | 547 | 785 | ... |
| ... | ... | Islington Medical Mission ... | ... | 14,095 | 911 | 660 | 492 | 104 | 1,256 | ... |
| ... | ... | St. Pancras and Northern ... | ... | 8,063 | 1,286 | 269 | ... | 941 | 1,210 | 170 |
| ... | ... | Stamford Hill, &c. ... ... | ... | 17,728 | 656 | 403 | 157 | ... | 560 | ... |
| 1 997 | 1,637 | | 16,763 | 532,421 | 204,591 | 98,822 | 25,566 | 71,243 | 195,631 | 33 941 |

### STRATFORD AND EAST-END DISTRICT.

**Comprising Bethnal Green, Tower Hamlets, West Ham, Whitechapel, Hackney, Stepney, Limehouse, Poplar, and the East.**

| No. of Beds. | No. of Beds Daily Occupied. | Hospitals. | In-patients | Out-patient Attendances. | Total Expenditure. | Income. Charitable. | Income. Proprietary. | Income. Patients' Payments. | Total Income. | Legacies not included in preceding column. |
|---|---|---|---|---|---|---|---|---|---|---|
| | | | | | £ | £ | £ | £ | £ | £ |
| 1,003 | 793 | London ... ... ... ... | 15,313 | 341,189 | 191,979 | 84,153 | 45,903 | 15,478 | 145,534 | 24,494 |
| 32 | 30 | Mildmay Mission Hospital ... | 307 | 16,237 | 4,404 | 2,849 | 624 | 386 | 3,859 | 25 |
| 30 | 19 | Mildmay Memorial ... ... | 257 | ... | 2,576 | 1,117 | 983 | 272 | 2,372 | ... |
| 115 | 89 | Poplar ... ... ... ... | 1,908 | 66,339 | 13,895 | 13,840 | 3,466 | 1,961 | 19,267 | 2,768 |
| 50 | 39 | Walthamstow, &c. ... ... | 508 | 11,379 | 2,865 | 2,085 | 534 | 60 | 2,679 | 200 |
| 179 | 154 | Queen Mary's, &c. ... ... | 2,026 | 122,366 | 19,100 | 15,889 | 1,538 | 4,295 | 21,722 | 1,750 |
| 175 | 161 | City of London for Dis. of the Chest | 626 | 34,199 | 20,646 | 13,709 | 1,292 | 4,058 | 19,059 | 5,819 |
| 130 | 105 | East London, for Children ... | 1,381 | 58,961 | 13,319 | 11,595 | 2,126 | 71 | 13,792 | 110 |
| 43 | 40 | St. Mary's, Plaistow, for Children | 560 | 21,712 | 5,367 | 3,957 | 646 | 75 | 4,678 | 100 |
| 33 | 25 | East End Mothers' Home ... | 643 | 17,185 | 3,480 | 1,997 | 1,029 | 591 | 3,617 | 250 |
| 20 | 17 | Plaistow Maternity ... ... | 447 | 362,560 | 1,309 | 901 | 1 | 325 | 1,227 | 2 |
| 26 | 19 | Canning Town Cottage ... ... | 226 | 6,575 | 2,607 | 1,298 | 355 | 394 | 2,047 | ... |
| 17 | 9 | Passmore Edwards Cottage, T'lb'ry | 212 | 2,370 | 851 | 872 | 90 | 22 | 984 | ... |
| 25 | 13 | East Ham Cottage ... ... | 133 | 13,910 | 1,409 | 580 | 433 | 609 | 1,622 | ... |
| 1,878 | 1,513 | | 24,547 | 1,074,982 | 283,807 | 154,842 | 59,020 | 28,597 | 242,459 | 35,518 |
| | | **Dispensaries.** | | | | | | | | |
| ... | ... | All Saints', Buxton Street ... | ... | 1,487 | 102 | 97 | ... | ... | 97 | ... |
| ... | ... | Eastern ... ... ... ... | ... | 19,705 | 816 | 205 | 266 | 336 | 807 | ... |
| ... | ... | London ... ... ... ... | ... | 1,063 | 439 | 77 | 270 | ... | 347 | ... |
| ... | ... | Queen Adelaide's ... ... ... | ... | 9,511 | 568 | 360 | 352 | ... | 712 | 400 |
| 1,878 | 1,513 | | 24,547 | 1,106,748 | 285,732 | 155,581 | 59,908 | 28,933 | 244,422 | 35,918 |

### KENSINGTON AND WEST DISTRICT.

**Comprising Kensington, Paddington, Bayswater, Kilburn, Chelsea, Brompton, Fulham, Hammersmith, Chiswick, Brentford, Acton, Ealing, &c.**

| No. of Beds. | No. of Beds Daily Occupied. | Hospitals. | In-patients | Out-patient Attendances. | Total Expenditure. | Income. Charitable. | Income. Proprietary. | Income. Patients' Payments. | Total Income. | Legacies not included in preceding column. |
|---|---|---|---|---|---|---|---|---|---|---|
| | | | | | £ | £ | £ | £ | £ | £ |
| 341 | 307 | St. George's ... ... ... | 4,092 | 117,184 | 54,005 | 14,002 | 13,003 | 6,715 | 33,720 | 13,788 |
| 298 | 251 | St. Mary's ... ... ... ... | 2,861 | 97,763 | 41,825 | 12,903 | 15,265 | 6,577 | 34,745 | 6,573 |
| 168 | 165 | West London ... ... ... | 2,268 | 155,870 | 25,912 | 16,082 | 1,170 | 4,168 | 21,420 | 2,278 |
| 483 | 433 | Hospital for Consumption ... | 1,469 | 29,422 | 53,493 | 22,747 | 4,493 | 20,027 | 47,267 | 7,635 |
| 40 | 38 | Belgrave, for Children ... ... | 647 | 34,775 | 5,768 | 4,402 | 845 | 340 | 5,587 | 500 |
| 79 | 79 | Cheyne, for Sick & Incurable Chldn | 52 | ... | 5,248 | 1,689 | 1,575 | 591 | 3,855 | 2,662 |
| 22 | 19 | Kensington, General ... ... | 305 | 22,097 | 2,917 | 2,066 | 44 | 1,004 | 3,114 | 15 |
| 11 | 9 | Kensington, for Children .. | 155 | 8,524 | 1,243 | 1,901 | 248 | ... | 2,149 | ... |
| 46 | 35 | Paddington Green, for Children | 742 | 41,356 | 7,053 | 7,492 | 1,182 | 484 | 9,158 | 878 |
| 155 | 124 | Victoria, for Children ... ... | 1,189 | 64,809 | 11,377 | 8,443 | 2,608 | 460 | 11,511 | 115 |
| 57 | 47 | Chelsea, for Women ... ... | 624 | 7,654 | 9,207 | 4,088 | 1,224 | 2,319 | 7,631 | 137 |
| 127 | 96 | Cancer ... ... ... ... | 721 | 20,310 | 22,942 | 2,982 | 10,309 | ... | 13,291 | 8,348 |
| 145 | 110 | Female Lock ... ... ... | 613 | ... | 11,081 | 6,116 | 679 | 4,233 | 11,028 | 2,852 |
| 24 | 21 | Banstead Surgical Home ... | 131 | ... | 1,048 | 446 | 86 | 545 | 1,077 | ... |
| 35 | 31 | Acton Cottage ... ... ... | 299 | 8,827 | 2,538 | 1,744 | 200 | 789 | 2,733 | 100 |
| 70 | 50 | Ealing Cottage ... ... ... | 655 | ... | 4,558 | 2,814 | 536 | 1,977 | 5,327 | 1,015 |
| 18 | 11 | Epsom and Ewell Cottage ... | 190 | ... | 1,032 | 470 | 63 | 328 | 861 | 510 |
| 27 | 22 | Hounslow Cottage ... ... | 301 | 3,638 | 1,732 | 824 | 343 | 629 | 1,796 | 1,250 |
| 40 | 39 | Reigate and Redhill Cottage ... | 695 | ... | 3,519 | 1,984 | 281 | 665 | 2,930 | ... |
| 24 | 20 | Teddington Cottage ... ... | 272 | ... | 1,400 | 1,052 | 368 | 115 | 1,535 | ... |
| 30 | 30 | Wimbledon Cottage ... ... | 473 | ... | 2,610 | 1,412 | 405 | 1,633 | 3,450 | 145 |
| 35 | 31 | Wimbledon, Nelson Hospital ... | 531 | 2,443 | 2,633 | 1,400 | 318 | 661 | 2,379 | 100 |
| 2,275 | 1,968 | | 19,285 | 614,672 | 273,141 | 117,059 | 55,245 | 54,260 | 226,564 | 48,901 |
| | | **Dispensaries.** | | | | | | | | |
| ... | ... | Kilburn, Maida Vale ... ... | ... | 5,423 | 372 | 237 | 30 | ... | 267 | ... |
| ... | ... | Kilburn Provident ... ... | ... | 9,427 | 1,045 | 81 | 23 | 954 | 1,058 | ... |
| ... | ... | Notting Hill Provident ... ... | ... | 5,479 | 477 | 82 | 26 | 373 | 481 | ... |
| ... | ... | Paddington Provident ... ... | ... | 3,883 | 230 | 90 | 1 | 140 | 231 | ... |
| ... | ... | Royal Pimlico Provident... ... | ... | 5,660 | 354 | 177 | 81 | 46 | 304 | ... |
| ... | ... | Westbourne Provident ... ... | ... | 2,044 | 190 | 31 | 5 | 142 | 178 | ... |
| 2,275 | 1,968 | | 19,285 | 646,588 | 275,809 | 117,757 | 55,411 | 55,915 | 229,083 | 48,901 |

### NEWINGTON AND SOUTH DISTRICT.

**Comprising Battersea, Wandsworth, Tooting, Balham, Streatham, Brixton, Lambeth, Newington, Southwark, Bermondsey, Camberwell, Greenwich, Deptford, Lewisham, Blackheath, Woolwich, &c.**

| No. of Beds. | No. of Beds Daily Occupied. | HOSPITALS. | In-patients. | Out-patient Attendances. | Total Expenditure. | Income. | | | Total Income. | Legacies not included in preceding column. |
|---|---|---|---|---|---|---|---|---|---|---|
| | | | | | | Charitable. | Proprietary. | Patients' Payments. | | |
| | | | | | £ | £ | £ | £ | £ | £ |
| 643 | 546 | Guy's ... ... ... ... | 8,386 | 385,433 | 105,585 | 19,493 | 60,321 | 5,226 | 85,040 | 7,374 |
| 719 | 565 | King's College ... ... ... | 8,166 | 114,593 | 80,794 | 26,736 | 16,763 | 31,674 | 75,173 | 2,821 |
| 18 | 10 | Phillips' Memorial Homœopathic | 165 | 2,039 | 1,403 | 456 | 383 | 445 | 1,284 | 10 |
| 76 | 72 | Miller ... ... ... ... | 1,552 | 114,245 | 13,001 | 10,629 | 2,450 | 1,088 | 14,167 | 1,008 |
| 62 | 52 | St. John's, Lewisham ... ... | 407 | 12,681 | 5,231 | 2,144 | 710 | 1,751 | 4,605 | 20 |
| 1,014 | 876 | St. Thomas's ... ... ... | 9,780 | 242,686 | 129 831 | 4,423 | 36,868 | 70,646 | 111,937 | 13,153 |
| ... | ... | Dreadnought Seamen's Hospital* | ... | ... | ... | ... | ... | ... | ... | ... |
| 60 | 52 | Bolingbroke Hospital ... ... | 774 | 25,091 | 8,356 | 3,228 | 1,113 | 1,651 | 5,992 | ... |
| 76 | 43 | Evelina, for Children ... ... | 950 | 33,657 | 9,021 | 3,406 | 4,534 | 258 | 8,198 | 1,225 |
| 57 | 44 | Home for Sick Children ... ... | 532 | 5,609 | 3,757 | 3,738 | 402 | 649 | 4,789 | 60 |
| 14 | 10 | British Home for Mothers and Babies ... ... ... ... | 203 | 2,350 | 1,404 | 875 | 443 | 508 | 1,826 | ... |
| 36 | 32 | General Lying-In... ... ... | 873 | 10,215 | 7,696 | 4,219 | 2,960 | 190 | 7,369 | ... |
| 50 | 37 | Clapham Maternity & Dispensary | 915 | 3,732 | 3,586 | 855 | 760 | 2,127 | 3,742 | ... |
| 67 | 63 | South London, for Women ... | 913 | 28,248 | 10,251 | 6,210 | 2,237 | 3,174 | 11,621 | ... |
| 118 | 88 | Royal Waterloo ... ... ... | 873 | 16,710 | 10,480 | 8,768 | 2,471 | 4 | 11,243 | 871 |
| 42 | 40 | Royal Eye ... ... ... ... | 543 | 56,097 | 5,963 | 2,450 | 1,184 | 1,897 | 5,531 | 2,318 |
| 28 | 13 | Beckenham Cottage ... ... | 301 | 1,172 | 1,823 | 1 515 | 31 | 562 | 2,108 | 90 |
| 26 | 22 | Blackheath Cottage ... ... | 239 | 895 | 2,175 | 1,167 | 283 | 500 | 1,950 | 90 |
| 42 | 15 | Bromley Cottage ... ... ... | 289 | 238 | 2,096 | 1,066 | 850 | 402 | 2,318 | ... |
| 27 | 21 | Chislehurst, &c., Cottage ... | 316 | ... | 2,117 | 1,315 | 306 | 689 | 2,310 | ... |
| 22 | 12 | Eltham Cottage ... ... ... | 206 | ... | 1,173 | 745 | 146 | 369 | 1,260 | 100 |
| 11 | 6 | Sidcup Cottage ... ... ... | 103 | 117 | 818 | 614 | 35 | 217 | 866 | ... |
| 34 | 29 | Livingstone Cottage ... ... | 440 | ... | 1,945 | 1,620 | 133 | 134 | 1,887 | ... |
| 23 | 16 | Victoria Hospital, Kingston ... | 358 | ... | 1,467 | 720 | 296 | 325 | 1,341 | 680 |
| 3,265 | 2,664 | | 37,281 | 1,055,808 | 409,973 | 106,392 | 135,679 | 124,486 | 366,557 | 29,820 |
| | | DISPENSARIES. | | | | | | | | |
| ... | ... | Battersea Provident ... ... | ... | 79,800 | 3,340 | 147 | 59 | 3,145 | 3,351 | ... |
| ... | ... | Brixton, &c. ... ... ... | ... | 9,787 | 966 | 227 | 786 | 40 | 1,053 | 100 |
| ... | ... | Camberwell Provident ... ... | ... | 52,860 | 934 | 176 | 200 | 510 | 886 | ... |
| ... | ... | Clapham ... ... ... ... | ... | 8,751 | 439 | 261 | 112 | 178 | 551 | 300 |
| ... | ... | East Dulwich Provident ... | ... | 13,290 | 1,044 | 77 | 31 | 951 | 1,059 | ... |
| ... | ... | Forest Hill Provident ... ... | ... | 16,255 | 484 | 216 | 25 | 306 | 547 | 50 |
| ... | ... | Greenwich Provident ... ... | ... | 22,025 | 481 | 40 | 9 | 430 | 479 | ... |
| ... | ... | Wandsworth Common ... ... | ... | 1,380 | 165 | 11 | 12 | 142 | 165 | ... |
| ... | ... | Woolwich, &c., Provident ... | ... | 13,774 | 635 | 39 | ... | 564 | 603 | ... |
| 3,265 | 2664 | | 37,281 | 1,273,730 | 418,461 | 107,586 | 136,913 | 130,752 | 375,251 | 30,270 |

* Audit not yet completed. No figures received.

### THE MEDICAL CHARITIES OF LONDON.—A SUMMARY OF THE WORK DONE IN 1917.

It will be seen from the following summary that *One hundred and forty-eight thousand nine hundred and thirty-one* patients were admitted into the Voluntary Hospitals of London during the year 1917, and that the attendances in the Out-Patient Departments and Dispensaries numbered *Five millions four hundred and twenty-two thousand and twenty-one* at a cost of *£1,876,666*. The Ordinary Income amounted to *£1,678,408*, leaving a deficiency of *£198,258* on the year's work. The Legacies received in 1917 amounted to *£225,784*.

| No. of Beds. | No of Beds Daily Occupied. | HOSPITALS AND DISPENSARIES. | In-patients. | Out-patient Attendances. | Total Expenditure. | Income. | | | Total Income. | Legacies not included in preceding column. |
|---|---|---|---|---|---|---|---|---|---|---|
| | | | | | | Charitable. | Proprietary. | Patients' Payments. | | |
| | | | | | £ | £ | £ | £ | £ | £ |
| 3,265 | 2,664 | Newington and South District ... | 37,281 | 1,273,730 | 418,461 | 107,586 | 136,913 | 130,752 | 375,251 | 30,270 |
| 1,852 | 1,530 | City and East Central District... | 19,869 | 781,906 | 272,849 | 92,989 | 98,136 | 58,584 | 249,709 | 31,958 |
| 1,108 | 907 | Westminster District ... ... | 10,413 | 427,856 | 147,998 | 72,474 | 22,017 | 45,310 | 139,801 | 12,867 |
| 2,148 | 1,889 | St. Marylebone, &c., District ... | 20,773 | 652,772 | 271,226 | 117,464 | 51,817 | 75,230 | 244,511 | 31,929 |
| 2,275 | 1,968 | Kensington and West District ... | 19,285 | 646,588 | 275,809 | 117,757 | 55,411 | 55,915 | 229,083 | 48,901 |
| 1,997 | 1,637 | Islington & North-West District | 16,763 | 532,421 | 204,591 | 98,822 | 25,566 | 71,243 | 195,631 | 33,941 |
| 1,878 | 1,513 | Stratford and East-End District | 24,547 | 1,106,748 | 285,732 | 155,581 | 59,908 | 28,933 | 244,422 | 35,918 |
| 14,523 | 12,108 | | 148,931 | 5,422,021 | 1,876,666 | 762,673 | 449,768 | 465,967 | 1,678,408 | 225,784 |

